# Epstein-Barr Virus Associated Modulation of *Wnt* Pathway Is Not Dependent on Latent Membrane Protein-1

**DOI:** 10.1371/journal.pone.0003254

**Published:** 2008-09-22

**Authors:** Natasha Webb, Geoff Connolly, Judy Tellam, Alpha S. Yap, Rajiv Khanna

**Affiliations:** 1 Australian Centre for Vaccine Development and Tumour Immunology Laboratory, Division of Infectious Diseases and Immunology, Queensland Institute of Medical Research, Brisbane, Australia; 2 Institute of Molecular Biosciences, University of Queensland, Brisbane, Australia; Cambridge University, United Kingdom

## Abstract

Previous studies have indicated that Epstein-Barr virus (EBV) can modulate the *Wnt* pathway in virus-infected cells and this effect is mediated by EBV-encoded oncogene latent membrane protein 1 (LMP1). Here we have reassessed the role of LMP1 in regulating the expression of various mediators of the canonical *Wnt* cascade. Contradicting the previous finding, we found that the levels of E-cadherin, β-catenin, Glycogen Synthase Kinase 3ß (GSK3β), axin and α-catenin were not affected by the expression of LMP1 sequences from normal B cells or nasopharyngeal carcinoma. Moreover, we also show that LMP1 expression had no detectable effect on the E-cadherin and β-catenin interaction and did not induce transcriptional activation of β-catenin. Taken together these studies demonstrate that EBV-mediated activation of *Wnt* pathway is not dependent on the expression of LMP1.

## Introduction

The oncogenic potential of Epstein-Barr virus (EBV) is well recognized, and the virus is associated with a number of human malignancies, including Burkitt's lymphoma (BL) and nasopharyngeal carcinoma (NPC) [1]. Each of the EBV-associated malignancies is characterised by a unique viral and cellular phenotype. In most of the EBV-associated malignancies the viral gene expression is often restricted to a limited number of proteins. This limited gene expression is often considered as one of the most important factors in the pathogenesis and escape of these malignancies from immune control (see reviews [2], [3]. EBV-encoded oncogene latent membrane protein 1 (LMP1), has been recognised as one of most crucial latent proteins for EBV-mediated transformation of normal B cells and is uniquely able to induce malignant outgrowth and hyperplasia in transgenic mice [4]. Furthermore, LMP1 is also known to exhibit pleiotropic effects on the cellular phenotype of B cells which include induction of activation antigens, the expression of inhibitors of programmed cell death and NF-κB activation through the TRAF signalling pathway [5]–[7]. Previous studies have shown that LMP1 acts as a constitutively active receptor like molecule independent of the binding of a ligand [1], [8]. The transmembrane domains mediate oligomerization of LMP1 molecules in the plasma membrane, a prerequisite for LMP1 function [1], [9].

Over the last few years, there has been increasing evidence to suggest EBV is capable of modulating the *Wnt* pathway [10]–[13]. In particular, it has been suggested LMP1 expression can repress the expression of E-cadherin [14]–[16]. The current experiments reported here were undertaken to reassess the role of LMP1 in regulating the expression of E-cadherin and to further explore the mechanism by which LMP1 modulates the function of various mediators of the canonical *Wnt* cascade. Here we show that transient or stable expression of LMP1 sequences from normal B cells and NPC does not impair the expression of E-Cadherin and other mediators of the Wnt pathway. Furthermore, we also demonstrate that LMP1 expression in human cells had minimal effect on the interaction of E-cadherin and β-catenin thus no evidence of β-catenin-mediated transcriptional activation was observed.

## Results and Discussion

### Expression of Wnt pathway mediators in LMP1-positive cells

**Figure 1:**
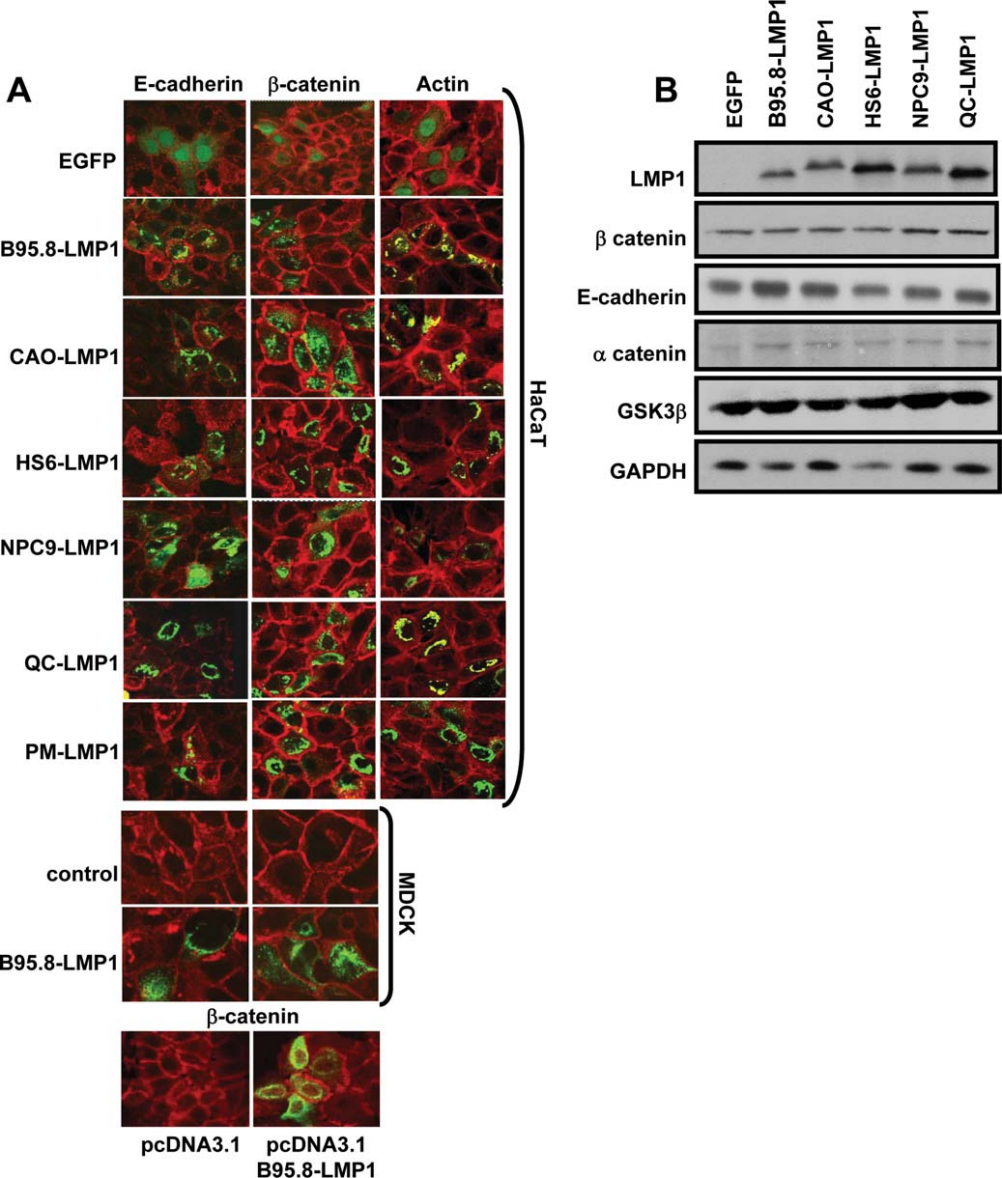
Panel A: Effect of LMP1 on the expression of E-cadherin, β-catenin and actin. HaCaT or MDCK cells were transiently transfected with expression vectors encoding LMP1 protein fused to EGFP. LMP1 sequences were derived from either the prototype B95.8 isolate, spontaneous LCLs (HS6, QC and PM) or NPC biopsies (CAO and NPC9). Following transfection, these cells were cultured for 36–48 h and then assessed for the expression of E-cadherin, β-catenin and actin using confocal microscopy. HaCaT cells transfected with pcDNA3.1 vector with or without B95.8-LMP1 were also assessed for β-catenin expression (bottom panels). Panel B: HEK293 cells transfected with various LMP1 sequences were also processed for SDS-PAGE and immunoblot analysis. Antibodies specific for β-catenin, E-cadherin, α-catenin, GSK3β were used to assess the level of expression each of these components in LMP1 or EGFP expressing cells. In addition, the level of LMP1 and GAPDH expression was also assessed in these cells. Representative data from one of the five different experiments is presented in this figure.

To explore the effect of LMP1 on other mediators of the Wnt pathway, we transiently transfected HaCaT and MDCK cells with expression vectors encoding LMP1-GFP or the control EGFP vector. These LMP1 sequences were either derived from the prototype B95.8 isolate, spontaneous LCLs (HS6, QC and PM) or NPC (NPC9 and CAO). After transfection, these cells were examined using confocal microscopy for the expression of E-cadherin, β-catenin or actin. Representative data from a series of experiments is presented in Figure 1 (panel A). In contrast to the previous studies, we observed very little difference in the expression of E-cadherin or β-catenin in LMP1 or EGFP-positive cells. Both HaCaT and MDCK cells showed minimal effect of LMP1 on the expression of E-cadherin, β-catenin. Interestingly, LMP1 sequences from both normal B cells or from NPC showed no effect on the expression of E-cadherin and β-catenin. On the other hand, we did noticed alteration in the organization of actin filaments in LMP1 expressing cells which is consistent with the previous studies published by Dawson and colleagues who also showed actin filament remodelling following LMP1 expression in 3T3 fibroblasts [17]. To ensure that the results described above were not influenced by the covalent linking of LMP1 with EGFP, we also expressed LMP1 protein in HaCaT cells without EGFP and then assessed the expression of β-catenin. Consistent with the data presented above, we observed no significant difference in the pattern of β-catenin expression in HaCaT cells transfected with either pcDNA3.1 (control) or pcDNA3.1 encoding B95.8-LMP1 (Fig. 1, Panel A).

**Figure 2:**
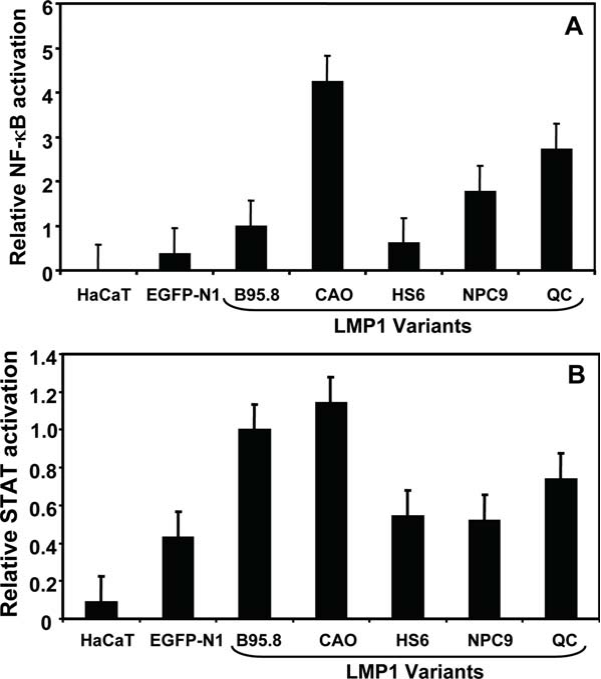
Effects of the LMP1 expression constructs on the activation of cellular signaling pathways. NFκB (panel A) and STAT (panel B) activation induced by B95-8-LMP1, HS6-LMP1, NPC9-LMP1, QC-LMP1 or CAO-LMP1 was assessed by quantitation of the luciferase produced from a co-transfected reporter plasmids, 3Enh.κB-ConALuc or GRR(5)-Luc. The data were normalized for transfection efficiency by measuring GFP-positive cells and then expressed relative to the activity obtained with the B95-8-LMP1 (100%) without subtracting the basal activity in control pEGFP-N1-transfected cells. Results are the mean and standard deviation of at least four separate experiments.

To further confirm these observations, we resolved the protein samples (normalised for GFP expression using FACS analysis) from these transfected cells on SDS-PAGE followed by immunoblotting. Data presented in Figure 1, panel B clearly demonstrate that the levels of E-cadherin and β-catenin were largely unaffected by the expression of LMP1. In addition, expression levels of other Wnt pathway mediators and potential modulators (α-catenin and GSK3β) was indistinguishable between LMP1 and EGFP-positive cells. It is important to point out that the lack of any modulation of Wnt pathway mediators by LMP1 in these experiments was not due to either low levels of LMP1 expression or the loss of LMP1-mediated signalling due to covalent linking of EGFP. All LMP1 expression vectors showed normal to high levels of LMP1 expression which was quite comparable to the levels seen in EBV-infected B cells. Furthermore, data presented in figure 2 clearly shows that expression vectors encoding LMP1 protein fused to GFP at the C-terminus are fully capable of activating NF-κB and STAT3 which is comparable to that seen with LMP1 protein without GFP [18]. It is interesting to note that LMP1 sequences displayed some differences with respect to their ability to activate NF-κB and STAT3, although this variation not particularly associated with any specific disease setting from which the LMP1 sequence was isolated.

**Figure 3:**
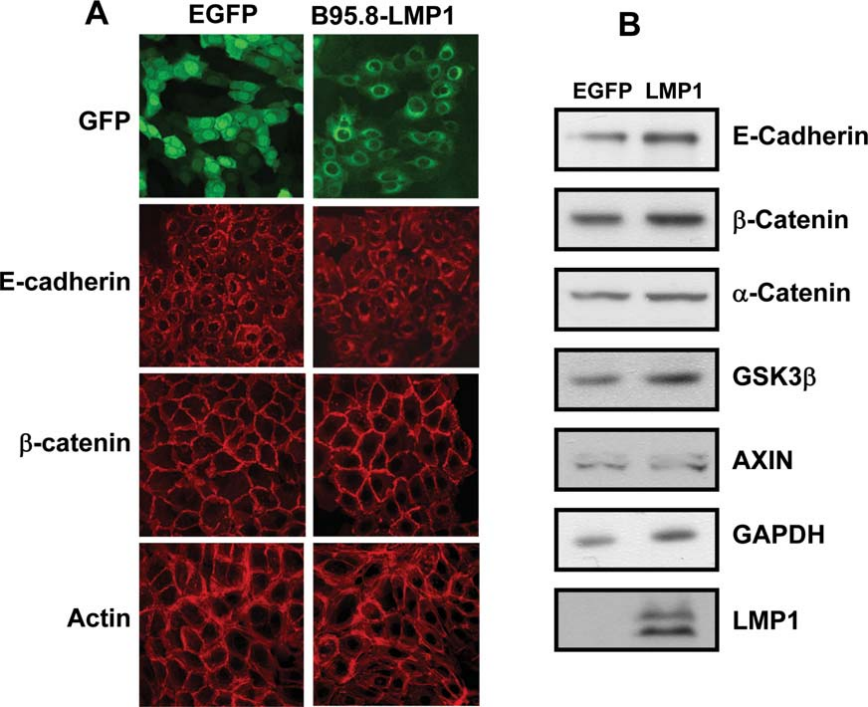
Effect of stable LMP1 expression on the canonical *Wnt* pathway mediators. HaCaT cells were transfected with expression vectors encoding LMP1 protein from the prototype B95.8 isolate. Following transfection these cells were cultured in growth medium supplemented with Geneticin for 3 weeks and stable transfectants isolated by FACSort. These stable transfectants were assessed for the expression of E-cadherin, β-catenin and actin using confocal microscopy (Panel A) and as well for α-catenin, GSK3β and Axin by SDS-PAGE and immunoblotting.

To ensure that these observations were not influenced by the transient expression of LMP1, we established stable transfectants of the HaCaT cell line expressing B95.8-LMP1 and EGFP. These cells were processed for E-cadherin, β-catenin and actin expression using confocal microscopy. Similar to data obtained with transient transfection, HaCaT cells stably expressing LMP1 showed minimal change in the expression of E-cadherin and β-catenin when compared to the cells expressing EGFP alone (Figure 3, panel A). Immunoblot analysis of protein samples from these cells also showed comparable levels of E-cadherin, β-catenin α-catenin, GSK3β, and axin in LMP1 and EGFP expressing cells (Figure 3, Panel B). Furthermore, analysis of β-catenin expression in the cytoplasmic and nuclear fraction of cells expressing LMP1 or EGFP revealed no significant difference (data not shown). Taken together, these data clearly demonstrate that LMP1 does not influence the expression of various mediators and potential modulators of the *Wnt* cascade and there is no evidence of accumulation of β-catenin either in the cytoplasm or nucleus following expression of LMP1.

### Effect of LMP1 on the interaction of E-Cadherin, β-catenin and α-catenin with the surface membrane

**Figure 4:**
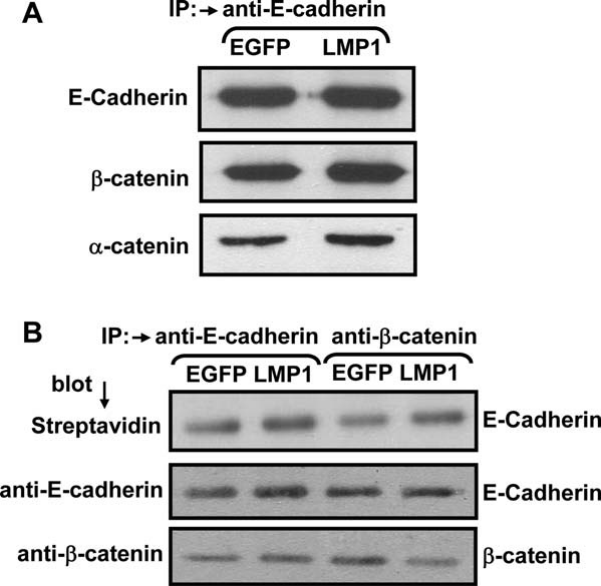
Effect of LMP1 expression on the interaction of E-cadherin and β-catenin. Two different methods were used to assess the effect of LMP1 on this interaction. Firstly, E-cadherin was immunoprecipated from the cells stably expressing either LMP1 or EGFP. These immunoprecipitates were then resolved on SDS-PAGE gel followed by immunoblotting (Panel A). These immunoblots were probed with antibodies specific for E-cadherin, β-catenin and α-catenin. In the second strategy, live HaCaT cells stably expressing either LMP1 or EGFP were initially surface labelled with biotin and then immunoprecipitated with anti- E-cadherin or anti-β-catenin antibodies. These immunoprecipitates were then resolved on SDS-PAGE gel followed by immunoblotting (Panel A). These immunoblots were probed with either streptavidin or antibodies specific for E-cadherin and β-catenin.

Although the data presented above clearly indicated that LMP1 does not influence the expression of individual components of the Wnt pathway, it is possible that LMP1 may disrupt the interaction of E-cadherin and β-catenin. A series of experiments were designed to immunoprecipitate E-cadherin and β-catenin complexes from LMP1 and EGFP expressing cells. In the first set of experiments, E-Cadherin was immunoprecipitated from the whole cell extract and then resolved on SDS-PAGE followed by immunoblotting. The immunoblots were probed with anti-E-cadherin, anti-β-catenin and anti- α-catenin antibodies. Data presented in Figure 4, panel A shows that LMP1 expression in HaCaT cells had very little effect on the interaction of E-cadherin, β-catenin and α-catenin. To further confirm these observations, we surface labelled LMP1 and EGFP-positive HaCaT cells with NHS-biotin and immunoprecipitated the biotin-labelled proteins with either anti-E-cadherin or anti-β-catenin antibodies. These immunoprecipitates were resolved on SDS-PAGE followed by immunoblotting with streptavidin, anti-E-cadherin or anti-β-catenin antibodies. Similar to the data presented in the panel A, we noticed very little effect of LMP1 expression on the interaction of E-Cadherin and β-catenin on the cell surface (Fig. 4, panel B).

**Figure 5:**
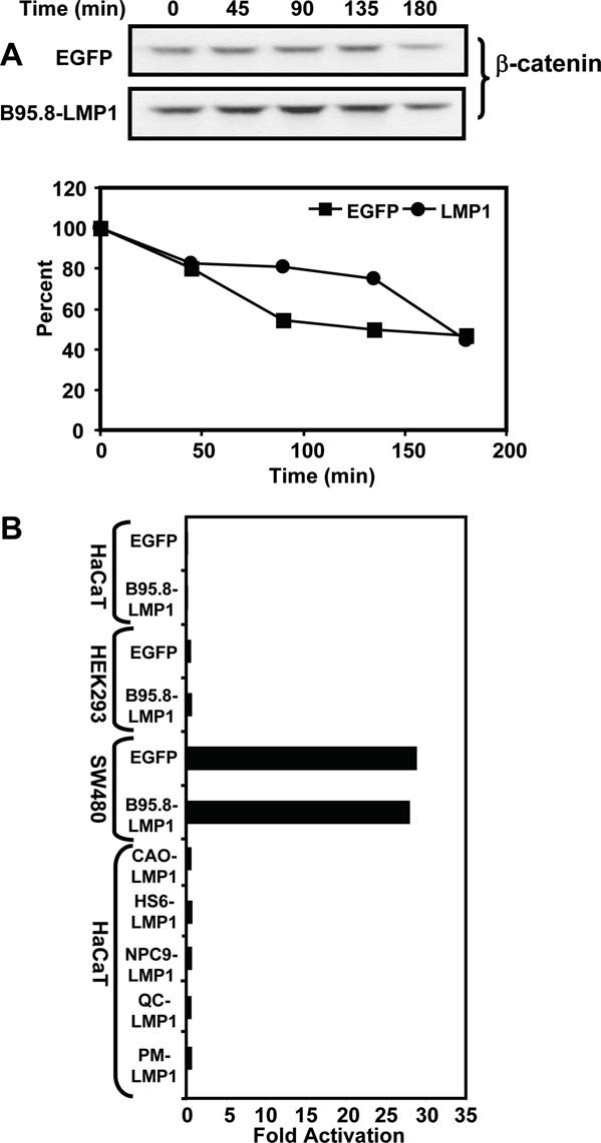
Panel A: Effect of LMP1 on the half-life of β-catenin. HaCaT cells expressing LMP1-GFP or EGFP pre-treated with cyclohexamide and then equal aliquots of cells were removed at time points 0 min, 45 min, 90 min, 135 min and 180 min; and lysed in RIPA buffer, resolved on SDS-polyacrylamide gel followed by immunoblotting with antibodies specific β-catenin. Protein bands densitometricaly analysed using Imagequant software. Panel B: Assessment of β-catenin transcriptional activity in LMP1 expressing cells. LMP1 or EGFP expressing cells (HaCaT, Hek293 or SW480) were co-transfected with TOPFlash or FOPFlash plasmids. These cells were used in luciferase reporter assays as described in the “Material and Methods” section. The TOPFlash luciferase activity produced by each sample is shown relative to the matching FOPFlash activity produced.

Previous studies have suggested that EBV-mediated activation of β-catenin involves stabilization of this protein which leads to β-catenin-mediated increased transcriptional activity. To explore the possibility that this effect may be mediated by LMP1, stable transfectants (LMP1 and EGFP-positive) were pretreated with cycloheximide to block fresh protein synthesis and protein samples collected at different time intervals. These samples were then resolved on SDS-PAGE followed by immunoblotting with the β-catenin-specific antibody. Data presented in Figure 5, panel A, shows no evidence of increased stabilization of β-catenin in LMP1 expressing cells. These experiments were repeated at least five times and we were unable to see any firm evidence of LMP1-medaited stabilization of β-catenin.

Another possible approach to test the stabilization of β-catenin is to assess downstream transcriptional activity mediated by this protein. It is now well established that stabilized β-catenin forms a complex with Tcf/lymphoid enhancer factor transcriptional factors and that this complex transactivates various cellular oncogenes (e.g. c-myc and cyclin D1) which play crucial role in cell transformation and tumour development [19], [20]. To investigate whether LMP1 expression results in β-catenin-mediated transcriptional activation of Tcf, we transfected HaCaT cells with EGFP or LMP1-GFP expression plasmids (B95.8, CAO, HS6, NPC9 or QC) in combination with Tcf reporter plasmids containing three copies of WT Tcf-binding site (TOPFLASH) and three copies of mutated site as a negative control (FOPFLASH) and used these cells in luciferase reporter assays. Representative data one of these experiments is presented in Figure 5, Panel B. Consistent with data presented in panel A, we observed no evidence of β-catenin-mediated transcriptional activity in LMP1-expressing cells.

These experiments were undertaken to reassess the role of LMP1 in modulating the canonical Wnt pathway. We have used human epithelial and keratinocyte cell lines to express different sequence variants of LMP1 and studied its effect on the regulation of E-cadherin and β-catenin interaction/function. The effect of LMP1 expression was assessed using both a transient and stable expression system. Confocal microscopic studies showed none of the LMP1 sequences (derived form either normal B cells or NPC) had any dramatic effect on the expression of E-Cadherin and β-catenin. Furthermore, we also found no effect of LMP1 on the interaction of E-Cadherin and β-catenin and the downstream β-catenin-mediated transcriptional activity. Based on this extensive and in depth analysis, we propose that it is unlikely that LMP1 plays any significant role in the modulation of the Wnt pathway. It is very difficult to precisely identify the reason for difference in the results described here and those described previously by other groups [14]–[16]. One possible reason might be that different cell lines respond differentially to LMP1 signalling. For example our studies were primarily based on human epithelial cell line HaCaT, while other groups have used canine epithelial cell line (MDCK). Furthermore, it is also possible that minor differences within the LMP1 sequences used by different groups may differentially impact on the expression of E-Cadherin and other mediators of Wnt pathway.

It is important to stress here that our studies do not refute a potential role of EBV in activating β-catenin and its transcriptional activity. It is possible that another EBV protein(s) play a crucial role in regulating β-catenin activity in virus-infected normal and malignant cells. Indeed, recent studies by Morrison and colleagues have shown that EBV-encoded Latent membrane protein 2A (LMP2A) activates β-catenin in epithelial cells through the PI3/AKt pathway [12], [21]. In this context, it is important to point out that LMP2A is consistently expressed in type II malignancies such as NPC where dysregulation of E-cadherin or β-catenin expression has been reported [21]–[23].

## Materials and Methods

### Expression Plasmids and Transfection

LMP1 sequences were amplified from prototype B95.8 isolate (referred to as B95.8-LMP1), NPC (referred to as NPC9-LMP1, CAO-LMP1) or spontaneous lymphoblastoid cell lines (LCL; HS6-LMP1, QC-LMP1, PM-LMP1) using sequence-specific primers and PCR and cloned in frame into the pEGFP-N1 vector (Clontech, Palo Alto, California). Amplified LMP1 sequences were ligated into the EcoR1 and BamHI sites of pEGFP-N1 in frame so as to express a fusion protein with the Green Fluorescent Protein (GFP) at the C-terminus. Human epithelial or keratinocyte cell lines (HaCaT, HEK293, SVMR6) or Madin-Darby canine kidney cell line (MDCK; ATCC no. CCL-34) were transfected with either pEGFP-N1 or LMP1-GFP expression vectors using lipofectamine 2000 (Invitrogen, Calsbad, California). In some experiments, an expression vector (pcDNA3.1) encoding full-length LMP1 without GFP fusion protein was also used These cells were cultured for 36–48 h in RPMI-1640 supplemented with 10% FCS (growth medium) and the transfection efficiency was assessed by the expression of EGFP using FACScalibur (Becton Dickinson, San Diego, California). In some cases transfected cells were cultured in growth medium supplemented with Geneticin (Invitrogen, Calsbad, California, 500 µg/ml) for 3 weeks and stable transfectants isolated by FACSort (Mo-Flo Fluoroscence Activated Cell Sorter, Dako Cytomation, Forte Collins, CO).

### Immunofluorescence

For immunofluorescence studies, LMP1 or EGFP expressing cells were seeded onto coverslips and cultured overnight in growth medium. After incubation, these cells were washed in PBS plus Calcium and Magnesium (PBSCM) and then fixed in 3% paraformaldehyde and permeabilised with 0.1% Saponin (Sigma Aldrich, St Louis, MO) in 5% FCS/PBSCM. After fixation, cells were incubated with monoclonal or polyclonal antibodies specific for LMP1 (Dako, Carpinteria, CA), β-Catenin (BD Transduction Laboratories, San Jose, CA) and E-Cadherin (BD Transduction Laboratories, San Jose, CA), and TRITC conjugated anti-Phalloidin antibody (Sigma Aldrich, St Louis, MO) in 5% FCS/PBSCM and incubated on the cells for 1 hour at room temperature. After washing in 0.1% Saponin/PBSCM, cells were incubated with anti-mouse Cy3 (Jackson ImmunoResearch Laboratories, Baltimore Pike, PA). Finally the cells were washed and examined by confocal microscopy (Leica TCS SP2; Leica, Mannheim, Germany).

### Surface Labeling, Immunoprecipitation and Immunoblotting

Monolayers of HaCaT cells stably expressing LMP1 or EGFP were washed with cold PBS (pH 8.0) and labelled with 2 mM EZ-Link Sulfo-NHS-Biotin Reagent (Pierce, Rockford, IL). The reaction was quenched with PBS and 100 mM Glycine prior to solubilisation with modified radioimmunoprecipitation (RIPA) buffer (Upstate Inc. Waltham, MA). These cell extracts were initially incubated with E-Cadherin-specific antibody for 2.5 hours followed by an overnight incubation with Protein G Sepharose beads (Roche Diagnostics, Mannheim, Germany). After incubation, beads were extensively washed with RIPA buffer and protein samples resolved using standard SDS-PAGE, transferred to nitrocellulose membrane and incubated with HRP-conjugated streptavidin (Chemicon, Temecula, CA), and antibodies specific for E-Cadherin, β-catenin or α-catenin (BD transduction laboratories, San Jose, CA),. Protein bands were detected using Chemiluminescence Reagent Plus (PerkinElmer, Life Sciences, Boston, MA) and their intensity compared by densitometric analysis using Imagequant software (Molecular Dynamics, Sunnyvale, CA). In some experiments protein samples from EGFP and LMP1-GFP expressing cells were directly resolved on the SDS-PAGE, transferred to nitrocellulose membrane and incubated with HRP-conjugated streptavidin, and antibodies specific for LMP1, E-Cadherin, β-catenin, α-catenin or GSK3β (Becton Dickson Transduction Laboratories, San Jose, CA), GAPDH (Ambion, Austin, TX) and Axin (Zymed Laboratories Inc. San Francisco, CA).

### Intracellular stability analysis of β-catenin

The human keratinocyte cell line HaCaT was transiently transfected with expression constructs encoding LMP1-GFP or EGFP as described above. At 30 h posttransfection, cyclohexamide (50 µg/ml) was added to 1×10^6^ cells and equal aliquots of cells were removed at time points 0 min, 45 min, 90 min, 135 min and 180 min; and lysed in RIPA buffer, resolved on SDS-polyacrylamide gel, transferred to nitrocellulose membrane and incubated with antibodies specific β-catenin. Protein bands were detected using Chemiluminescence Reagent Plus densitometricaly analysis using Imagequant software.

### Analysis of β-catenin transcription activity

Approximately, 10^6^ HaCaT cells were co-transfected with TOPFlash or FOPFlash (Upstate Biotechnology, Lake Placid, NY). in combination with EGFP or LMP1-GFP expression plasmids (B95.8, CAO, HS6, NPC9 or QC) at a ratio of 1∶2. After incubation for 24 h, a small aliquot of these cells was analysed for GFP expression by FACSCalibur and cell numbers were accordingly standardised based on GFP positive cells. These cells were used in luciferase reporter assays according to the manufacturer's instructions (Promega, Madison, WI). The TOPFlash luciferase activity produced by each sample is shown relative to the matching FOPFlash activity produced.

### Gene transfection and luciferase reporter assay

HaCaT cells were grown to late-log phase of cell growth prior to co-transfection. On the day of transfection, luciferase reporter plasmids for either NF-κB (3.Enh-Luc) or STAT (pGRR5-Luc) (Fielding et al., 2001) were combined with LMP1-GFP expression plasmids (B95–8, CAO, HS6, NPC9 or QC) and pEGFP-N1 expression vector in a 1∶2 ratio. Electroporation was used for the STAT co-transfections where 9 µg of total DNA was used for 5×10^6^ cells, and 0.8 µg total DNA was added to 2×10^5^ cells when using Effectene™ (Qiagen) for the NF-κB co-transfections. After growth for 24 hours in RPMI medium supplemented with antibiotics and 10% FCS, cells were harvested and resuspended in PBS supplemented with 2% FCS. To measure successful transfection a small aliquot was taken for FACScan (Becton Dickson) analysis of GFP expression, and the volume remaining was subsequently standardized to the sample of lowest efficiency. The transfection efficiencies were generally between 40–50%. To report the transcription factor activity, the cells were pelleted and lysed with 120 µl of Cell Culture Lysis Reagent (Promega) and the luciferase activity measured on 20 µl triplicates by addition of Luciferase Assay Reagent (Promega). The luciferase activity produced by each sample was calculated relative to the activity produced by the prototype B95-8-LMP1.
